# N-acetylcysteine Pharmacology and Applications in Rare Diseases—Repurposing an Old Antioxidant

**DOI:** 10.3390/antiox12071316

**Published:** 2023-06-21

**Authors:** Siddhee A. Sahasrabudhe, Marcia R. Terluk, Reena V. Kartha

**Affiliations:** Center for Orphan Drug Research, Department of Experimental and Clinical Pharmacology, Rm 4-214, McGuire Translational Research Facility, 2001 6th St. SE, College of Pharmacy, University of Minnesota, Minneapolis, MN 55455, USA

**Keywords:** antioxidant, pharmacology, repurposing, rare diseases, mechanism of action

## Abstract

N-acetylcysteine (NAC), a precursor of cysteine and, thereby, glutathione (GSH), acts as an antioxidant through a variety of mechanisms, including oxidant scavenging, GSH replenishment, antioxidant signaling, etc. Owing to the variety of proposed targets, NAC has a long history of use as a prescription product and in wide-ranging applications that are off-label as an over-the-counter (OTC) product. Despite its discovery in the early 1960s and its development for various indications, systematic clinical pharmacology explorations of NAC pharmacokinetics (PK), pharmacodynamic targets, drug interactions, and dose-ranging are sorely limited. Although there are anecdotal instances of NAC benefits in a variety of diseases, a comprehensive review of the use of NAC in rare diseases does not exist. In this review, we attempt to summarize the existing literature focused on NAC explorations in rare diseases targeting mitochondrial dysfunction along with the history of NAC usage, approved indications, mechanisms of action, safety, and PK characterization. Further, we introduce the research currently underway on other structural derivatives of NAC and acknowledge the continuum of efforts through pre-clinical and clinical research to facilitate further therapeutic development of NAC or its derivatives for rare diseases.

## 1. Introduction

N-acetylcysteine (NAC) is an acetylated analog of the endogenous semi-essential amino acid cysteine [1]. Cysteine serves as a precursor for synthesis of a remarkable array of molecules, one of which is the thiol-containing tripeptide glutathione (GSH) [1,2]. The formation of γ-glutamyl-L-cysteine—a dipeptide product of glutamate and cysteine ligation—catalyzed by glutamate-cysteine ligase is a rate-limiting step in the synthesis of GSH [3]. GSH is an important component of oxidation-reduction balance within the cells of human body. An imbalance between endogenous antioxidant defense systems and excessive production of reactive oxygen species (ROS) causes a shift in the cellular redox state towards an oxidizing state. This state known as oxidative stress; is also relevant in neurological and psychiatric diseases, as the brain is considered especially vulnerable to oxidative damage for several reasons, including its high oxygen utilization and hence generation of ROS, its modest antioxidant defenses, its lipid-rich neuronal circuitry that provides ready substrates for oxidation, the redox potential of certain neurotransmitters, and the presence of metals such as iron and copper essential for redox catalysis [4]. Coupled with the potential of self-perpetuating damage through neuronal atrophy and neuroinflammation, the oxidative stress mechanisms are plausible in the pathophysiology of many brain-related diseases [4]. Oxidative stress mechanisms have also been implicated in affective disorders [5]. It is not surprising that NAC has been studied extensively in neurodegenerative and psychiatric disorders, albeit with contradicting results [6,7]. Redox imbalance; characterized by antioxidant depletion and the accumulation of ROS and manifested into common themes such as neuronal damage, neurodegeneration, or inflammation, is implicated in the pathophysiology of many rare (e.g., amyotrophic lateral sclerosis (ALS), Huntington’s disease), and common diseases (e.g., Alzheimer’s disease (AD), Parkinson’s disease (PD)) [3,8,9]. Thus, it is hypothesized that the treatments that can directly or indirectly enhance the antioxidant defenses may provide health benefits in these seemingly divergent yet connected diseases [9]. Although it is reasonable to expect that supplementation of cysteine or GSH as such would serve to enhance the antioxidant pool; precursors and derivatives such as NAC are preferred due to their ease of formulation, stability, and superior attributes relevant to palatability and odor [7].

Oxidative stress damages the cell components and leads to altered functions [10]. The primary ROS production occurs in the mitochondria during the synthesis of ATP through aerobic respiration, wherein the transfer of electrons through the mitochondrial electron transport system generates anion superoxide. This process causes leakage of anion superoxide, which is converted to hydrogen peroxide (H_2_O_2_) through the action of superoxide dismutase and then detoxified by antioxidant enzymes such as catalase and glutathione peroxidase [11]. In addition to contributing to lipid metabolism, peroxisomes are metabolic organelles that generate ROS as byproducts of aerobic metabolism, which can impact cellular redox status. Peroxisomal metabolism and oxidative stress are closely linked and have been implicated in various human diseases [12]. Furthermore, the endoplasmic reticulum (ER) is another cellular source for ROS due to alterations in ER-regulated protein folding pathways [13]. Several studies have implicated redox imbalance, oxidative stress, and depletion of GSH in many pathological conditions, where unresolved cellular damage caused by the effects of ROS can result in cell death or drive the development of various diseases, including rare diseases—and treating this imbalance using an agent such as NAC can be beneficial [9,14].

In this review, we present a unique overview of the origin of NAC, its diverse applications, mechanisms of action, investigational uses in rare diseases, and relevant pharmacology that lie at the foundation of these uses. We also provide an overview of the safety of NAC and recent advances in the research with molecules that are structurally similar to NAC.

### 1.1. Origin of NAC and Its Mucolytic Application

**Figure 1:**
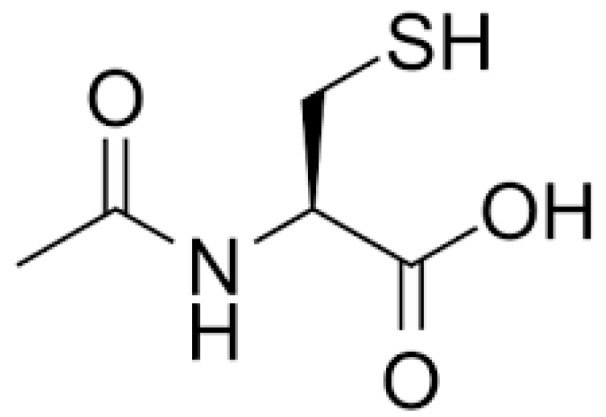
Chemical structure of N-acetylcysteine.

The earliest clinical development of NAC was for providing relief in muco-pulmonary ailments. Details such as the origin, synthesis, and application of NAC can be found in the patent “Mucolytic-N-acylated Sulfhydryl Compositions and Process for Treating Animal Mucus” filed for its mucolytic application [15]. As a mucolytic, NAC targeted an unmet need at the time when various treatment modalities such as vasoconstrictors, bronchodilators, surfactants, antibiotics, antihistamines, and proteolytic enzymes were being used for broncho-pulmonary ailments; however, no other product specifically reduced the viscosity of mucus accumulations. The free-sulfhydryl group in NAC (Figure 1) was demonstrated to disrupt mucus disulfide bonds, thereby causing a significant loss of viscosity in the mucus [16]. In contrast, other sulfur-containing compounds without the free-sulfhydryl group showed little to no effect on the viscosity of the mucus [16,17]. Early clinical observations attested to the clinical utility of NAC solution in decreasing the viscosity of tracheobronchial secretions in acute and chronic lung diseases while maintaining safety and, in some cases, improving symptoms associated with pulmonary occlusion [18,19]. Although approved by the Food and Drug Administration (FDA) for the treatment of cystic fibrosis (CF) complications, there is little high-quality published evidence on the therapeutic utility of NAC in CF [20]. In addition to CF complications, there are other FDA-approved broncho-pulmonary indications of NAC based on its mucus-thinning action (Table 1).

### 1.2. NAC as an Antidote of Acetaminophen Poisoning

While largely safe at the recommended doses, OTC analgesic acetaminophen (APAP; Tylenol^®^, paracetamol) in supra-therapeutic doses can be toxic and cause severe adverse outcomes, including death [21,22]. As a cysteine prodrug, NAC has been used as an antidote to reverse or limit GSH depletion and potential hepatic necrosis resulting from an overdose of acetaminophen. The oral solution of NAC was approved by the FDA for the treatment of acetaminophen overdose in 1978. In 2004, FDA approved the use of intravenous (IV) NAC as an antidote for the treatment of accidental and intentional acetaminophen overdose [23]. A systematic literature review and meta-analysis reports that when NAC was administered early (within 10 h of overdose) through IV or oral routes, the percentage of patients who developed a liver injury from acetaminophen poisoning was low, regardless of the route of administration [24]. NAC remains the standard of care for acetaminophen overdose and is included in the 2021 model list of essential medicines by the World Health Organization (WHO) (10% and 20% as an oral solution and 200 mg/mL in 10 mL volume for IV administration) [25]. Different dosage forms of NAC that are approved by the FDA for this indication are detailed in Table 1 [26].

**Table 1:** Approved Indications of NAC: Dosage Forms and Routes of Administration.

Dosage Form	Administration Route	Dose/Strength	Medical Condition/Therapy Type	Indication
Injectable	IV	200 mg/mL (6 g/30 mL)	Poisoning/antidote	Acetaminophen overdose reduction; prevention of acute hepatic injury; hepatic injury from repeated supra-therapeutic ingestion.
Effervescent tablet	Oral	500 mg 2.5 g		
Solution	Oral	10% 20%	Broncho-pulmonary disorders/Adjuvant therapy	Abnormal, viscid, inspissated mucous secretions in chronic [!] and acute [@] broncho-pulmonary disease. Pulmonary complications of cystic fibrosis; tracheostomy care; pulmonary complications associated with surgery; use during anesthesia; post-traumatic chest conditions; atelectasis due to mucous obstruction and diagnostic bronchial studies [#]
Solution	Inhalation	10% 20%		

[!] Chronic broncho-pulmonary disease: chronic emphysema, chronic emphysema with bronchitis, chronic asthmatic bronchitis, tuberculosis, bronchiectasis, and pulmonary amyloidosis of the lung. [@] Acute broncho-pulmonary disease: pneumonia, bronchitis, and tracheobronchitis. [#] Diagnostic bronchial studies: bronchograms, bronchospirometry, and bronchial wedge catheterization. This table is adapted from Šalmon et al., 2019 [26].

### 1.3. NAC as an OTC Product, a Nutraceutical, and a Dietary Supplement

For the past 30 years, NAC has been available in the US as an OTC dietary supplement. NAC is considered more resistant to oxidation compared to cysteine, offering ease of formulation as a solution without the repulsive odor. NAC is also more soluble and more stable compared to cysteine [27]. Although many OTC NAC products are available as multi-dose vials of NAC capsules, at least two companies, Zambon Pharma (Italy and US) and BioAdvantex (Canada and US), produce and package NAC in individual-dose packages to minimize exposure to air and thus oxidation [28]. Cetylev^®^, an approved oral NAC product, is also an individually packaged effervescent tablet, available in 500 mg or 2.5 g strength. Despite the long-standing use, the FDA’s recent decision excludes NAC from the dietary supplement definition under the Food Drug and Cosmetics (FD&C) Act (1938) [29].

## 2. NAC Pharmacology

The PK parameters of orally and intravenously administered NAC have been examined in several studies. It is important to note that the studies have measured different moieties of NAC and employed different compartmental or non-compartmental techniques for the estimation of PK parameters, limiting a direct comparison of the results. The PK parameters determined in select studies are summarized in Table 2. A summary of NAC PK is given below [30].

### 2.1. Absorption

After the administration of a single oral dose of 11 g of Cetylev^®^ (dissolved in 300 mL of water) to 29 healthy adult subjects, the mean of the maximum plasma concentration (C_max_) (CV%) was 26.5 (29) μg/mL and the mean (CV) of the area under the plasma concentration–time curve from time = 0 to infinity (AUC_inf_) was 186 (29) hr. μg/mL. The median (range) time to reach C_max_ (T_max_) was 2 (1 to 3.5) h.

### 2.2. Distribution

The steady-state volume of distribution (Vss) and the protein binding for NAC was reported to be 0.47 L/kg and 66–87%, respectively.

### 2.3. Metabolism

Acetylcysteine may form cysteine, disulfides, and conjugates in vivo (N,N′-diacetyl cysteine, N-acetylcysteine-cysteine, N-acetylcysteine-glutathione, N-acetylcysteine-protein, etc.). Published data indicate that after an oral dose of 35S-acetylcysteine, about 22% of the total radioactivity was excreted in the urine after 24 h. No metabolites were identified.

### 2.4. Elimination

After a single IV dose of acetylcysteine, the plasma concentration of total acetylcysteine declined in a poly-exponential decay manner with a mean terminal half-life (T1/2) of 5.6 h. The mean clearance (CL) for acetylcysteine was reported to be 0.11 L/h/kg, and renal CL constituted about 30% of the total CL [23].

### 2.5. Pharmacology in Special Populations

- Gender: No adequate information is available on whether there is any difference between the PK in males and females.
- Hepatic impairment: In persons with severe liver damage (Child–Pugh score of 7–13) or biliary cirrhosis (grade A and grade B, Child–Pugh score 5–7), the T1/2 increased by 80% and CL decreased by 30% compared to the healthy control group.
- Renal impairment: not enough information is available on the PK of NAC in persons with renal impairment. One study by Nolin et al. reports a reduction in NAC’s total CL by 90%, a seven-fold increase in AUC, and a 13-fold longer T1/2 in patients with end-stage renal disease (N = 24) compared to healthy individuals (N = 7) [40]. Given the contribution of nonrenal clearance to the total clearance of NAC, these results need to be independently replicated to assess the effect of renal impairment on NAC disposition [40].
- Pediatrics: The elimination of NAC is much slower (mean T1/2 of 11 h) compared to adults (5.6 h).
- Geriatrics: No adequate information is available.
- Drug–drug interactions (DDI): No DDI studies have been conducted.

## 3. NAC Safety

Mild and moderate gastrointestinal adverse events encompassing flatulence, nausea, vomiting, diarrhea, heartburn, and constipation were observed following oral NAC administration regardless of the dosage form [38,41]. The gastrointestinal adverse events were reported to be dose-dependent and those were resolved with titration of the NAC dose [42]. At a considerably smaller dose (200 mg/day administered orally as effervescent powder), Schmitt et al. reported no adverse findings in the hepatic enzyme panel and ultra-sensitive C-reactive protein [43]. Monti et al. reported no adverse events in a study evaluating a rather interesting dosing regimen, once a week IV NAC infusion (50 mg/kg) supplemented with oral NAC (500 mg BID) on non-infusion days [44]. In addition to the dose and route of administration, the adverse events may also be linked to the underlying physiological state, as PD participants on a 6000 mg/day dose of NAC reported worsening PD symptoms, whereas this dose was generally well-tolerated by the healthy group [36]. A recent review has extensively covered the safety of NAC administered by inhalation and in the treatment of pulmonary diseases [45]. The safety of NAC supplementation, or the lack thereof, in the treatment of cancer, although interesting, is beyond the scope of this report [46].

## 4. Key Mechanisms of Action

Multiple mechanisms have been proposed to explain the therapeutic benefits of NAC [47]. It is important to note that depending on the route of NAC administration, the dose, and the disease pathophysiology, some mechanisms become more plausible than others. Select mechanisms of NAC action are summarized below.

1. NAC as a reducing agent for disulfide bonds: This theory postulates that the beneficial effects of NAC are due, at least in part, to its capacity to reduce extracellular and intracellular disulfide bonds, making available free pools of bio-thiols such as cysteine.
2. The oxidant-scavenger theory of NAC action suggests that the free sulfhydryl group in NAC is an effective scavenger of one- and two-electron oxidants, such as H_2_O_2_, hypochlorous acid (HOCl), or hydroxyl radicals (•OH).
3. The NAC in GSH replenishment theory proposes that NAC acts as a prodrug for cysteine, which in turn boosts GSH synthesis.
4. NAC is also proposed to have anti-inflammatory properties that can be a direct action or attributed to its antioxidant activity.
5. The role of NAC in providing cystine, which can then be exchanged for glutamate in the brain’s glial cells, is an important theory underlying its use in the impulse-control spectrum of disorders.
6. NAC, as a pharmacological chaperone, highlights the ability of small-molecule NAC to assist the activity of deficient or otherwise misfolded proteins.

### 4.1. NAC as a Reducing Agent for Disulfide Bonds

The application of NAC as a mucolytic is based on its reducing effect. NAC was shown to reduce the disulfide bonds in the mucus, thereby liquefying it and providing relief for many muco-pulmonary diseases. As cysteine-rich molecules, mucus mucins form disulfide bonds that amass mucus into aggregates, stabilize the lobules, and increase their viscosity [48]. The reducing action of NAC on the mucus is highly plausible in the context of the earlier clinical studies [16,17,49] that applied a 20% (*w*/*v*) NAC solution (1.22 M) as an aerosol or by intratracheal instillations directly in the proximity of the mucus aggregates.

The postulated activity of disulfide bond reduction by NAC would take place through a direct thiol–disulfide exchange reaction to degrade the covalent bond(s), thus liberating free thiols such as cysteine. Two studies, one performed in vitro in plasma and another in vivo in mice using stable labeled NAC, were geared toward probing this mechanism of NAC action. Radtke et al. reported that in their incubation study, exogenous NAC added to the human plasma decreases cysteine–protein binding in a concentration-dependent manner [50]. There is a paucity of reports demonstrating the reducing action of NAC at clinically plausible concentrations. Therefore, this report by Radtke et al. [50] is significant, as their study was conducted at clinically observed concentrations of NAC (10–100 µg/mL, following a 70 mg/kg NAC dose in boys with adrenoleukodystrophy (ALD) undergoing hematopoietic stem cell transplant) [51]. Their finding of increased unbound cysteine in the plasma showed that NAC was able to liberate cysteine from its plasma-protein-binding sites [50]. In a subsequent study, Zhou et al. aimed to identify the role of NAC in GSH biosynthesis in mice using a stable-isotope-labeled tracer. Here, we observed that labeled NAC predominantly increased the unlabeled-GSH pool, suggesting that NAC increased GSH via indirect pathways. NAC can increase cysteine by thiol exchange reactions with plasma/tissue-protein-bound cysteine, thereby resulting in increased unlabeled GSH [52].

### 4.2. The Oxidant-Scavenger Action of NAC—Directly or Indirectly via Antioxidant Signaling

The theory that postulates NAC as a direct scavenger of reactive oxidant molecules is supported by fewer studies and less reported throughout the literature compared to the other proposed mechanisms of NAC action. There have been reports of oxidant-scavenging action of NAC in vitro [53] and in vivo [54]; however, the direct evidence through reaction catalysis rates is far from convincing. In particular, the critical parameter in the reaction kinetics is the rate of the reaction between NAC and various oxidants, which is fairly low (for H_2_O_2_, ~0.85 M^−1^ s^−1^; for •OH ~1010 M^−1^ s^−1^; for superoxide radical, undetectable) or, at best, comparable to other endogenous antioxidants, making a potential contribution of NAC irrelevant [47]. However, few reports support the elevation of antioxidant proteins such as HO-1 and catalase following clinical treatment with NAC, denoting that there may be a direct or even indirect oxidant-scavenging action of NAC [36,55]. More research directed at probing the specifics of the reaction mechanics, such as tracking the reaction products through the use of radio-labelled compounds, may better elucidate this action.

### 4.3. The Glutathione-Replenishment Action of NAC

The deacetylation of NAC into cysteine, which then leads to increased GSH biosynthesis, lies at the heart of NAC use in acetaminophen overdose [47,56]. Overall, the idea of using NAC as a prodrug of cysteine to replenish the GSH levels and thereby exert a protective effect against GSH-depleting xenobiotics is well-supported by the literature [57,58,59]. However, mechanistic studies that directly demonstrate that the positive effects of NAC depend on increased GSH biosynthesis are lacking.

Whether NAC is effective in replenishing GSH regardless of GSH deficiency is somewhat controversial. Few report the ineffectiveness of NAC in elevating GSH levels under normal conditions [60,61]. Others report oral NAC to increase the blood-cell GSH only for individuals with severely low GSH at the baseline, prior to receiving NAC treatment [62,63]. The lack of an increase in GSH levels in people with normal baseline GSH, despite NAC treatment and an abundance of cysteine, could be explained as occurring through a negative feedback mechanism that regulates the GSH biosynthesis pathway [64]. In contrast, Holmay et al. demonstrated a 34% increase in brain GSH in real time, even in healthy participants, following an IV infusion of NAC at 150 mg/kg dosage [65]. They observed that while the blood GSH/GSSG increased at approximately 1 h post-infusion, the brain GSH peaked approximately 1.5 h after the infusion [65]. Along similar lines, Coles et al. reported that they observed a significant increase in the peripheral GSH/GSSG and catalase on treatment with oral NAC in patients with PD who did not have elevated oxidative stress at baseline [36]. However, in their study, the increase in the peripheral antioxidant capacity did not translate into a meaningful decrease in peripheral measurements of oxidative damage (MDA, 4-HNE) [36]. In addition to the blood, some clinical studies have considered the modulation of GSH levels in the circulating blood cells and some body fluids (e.g., bronchoalveolar lavage and epithelial lining fluid in lungs) as a pharmacodynamic response to NAC [66]. The effect of acute or chronic oral NAC to improve GSH in tissues including the liver, kidneys, skin, and brain has been evaluated in several animal models of physiologic and pathologic conditions [66]. A recent review also summarizes the evidence that GSH supplementation leads to improved outcomes in chronic conditions such as metabolic and neurodegenerative diseases [67].

The discussion of GSH replenishment would be incomplete without acknowledging the role of genes regulating the endogenous antioxidant defense systems. The endogenous antioxidant defenses, including GSH synthesis, can be regulated by the transcription factor nuclear factor erythroid 2–related factor 2 (Nrf2), which plays an essential role in modulating the cellular response against oxidative stress and molecular damage [68,69]. In the absence of oxidative stress, Kelch-like ECH-associated protein 1 (Keap1) suppresses Nrf2 levels and its transcriptional activity by promoting its ubiquitination and proteasomal degradation. This downregulation of Nrf2 activity results in a suppression of the expression of antioxidant genes such as HO-1 and those involved in the thioredoxin and GSH systems [14]. Remarkably, Nrf2 modulates the mitochondrial antioxidant defenses, redox homeostasis, and bioenergetics by regulating the mitochondrial membrane potential, fatty acid oxidation, and the supply of substrates needed for mitochondrial respiration (such as NADH and FADH_2_/succinate) and ATP production [70].

The protective effect of NAC against oxidative stress can be attributed to the Nrf2 pathway, which increases GSH synthesis [71,72]. We have shown an induction in HO-1 signaling following high-dose NAC IV administration in boys with ALD undergoing HSCT [55]. The action of NAC in increasing GSH and thereby providing cytoprotection is likely to be through several direct or indirect pathways.

### 4.4. NAC as an Anti-Inflammatory Agent

The anti-inflammatory action of NAC is believed to be multifaceted. NAC can mitigate oxidative damage, which is often associated with inflammation. Additionally, NAC may directly modulate inflammatory signaling pathways, including nuclear factor-kappa B (NF-κB) and mitogen-activated protein kinases (MAPKs), leading to the suppression of pro-inflammatory cytokines and chemokines. Furthermore, NAC has been shown to inhibit the activation and recruitment of inflammatory cells such as neutrophils and macrophages, thus reducing tissue inflammation [73,74,75]. These combined mechanisms contribute to the ability of NAC to attenuate inflammation. However, these observations are largely from in vitro or ex vivo disease models and require further confirmation in clinical studies. It has been noted that the anti-inflammatory benefits of NAC in patients are dependent on the disease etiology and the dosage used [45].

### 4.5. Role of NAC in Regulation of Glutamate Homeostasis by Cystine–Glutamate Exchangers (System xc-)

NAC effects in the brain appear to occur via the active transport of cysteine and GSH across the blood–brain barrier (BBB) into the central nervous system [76]. Studies by our group using stable-isotope-labeled NAC isotopes confirm increased GSH levels in the brain [45]. Several animal studies have demonstrated that NAC can directly cross the BBB, although its mechanism requires further substantiation [36,52,65].

In addition, there are several postulated mechanisms through which NAC can exert its effect on neurochemicals without having to explicitly cross the BBB. In theory, NAC affects glutamatergic transmission in the CNS via a multistep indirect process. NAC is deacetylated into cysteine, which then dimerizes into cystine, an endogenous activator of system xc-, which transports cystine into glial cells in a 1:1 stoichiometry with glutamate transported into the extracellular environment. In other words, cystine is exchanged for glutamate in the glial cells. When extracellular glutamate levels are low, glial cells can influx cystine to trigger the efflux of glutamate, raising extracellular glutamate levels and ultimately restoring the tone on presynaptic mGluR2/3 auto receptors to dampen the neuronal release of synaptic glutamate. Through this mechanism, NAC has been proposed and tested as a possible pharmacotherapy for various neuropsychiatric conditions associated with the impulse-control spectrum of disorders, e.g., drug addiction, pathological gambling, psychotic mood, and anxiety disorders [77,78,79,80]. Dysregulation within the glutamate system has been implicated in these habit-based disorders, and there have been reports that suggest that NAC may provide clinical benefits in treating those disorders [81,82,83].

### 4.6. NAC as a Pharmacological Chaperone

NAC is hypothesized to bind and stabilize misfolded proteins and route them correctly within the cell, thereby facilitating their activity by acting as a pharmacological chaperone. A chaperone is usually a small molecule that restores the correct folding of mutant enzymes, facilitating their trafficking to the lysosomes and thus increasing the residual enzyme activity. This can result in a reduction in the signs and symptoms in patients with inherited disorders. In Pompe disease, a rare lysosomal disorder, NAC was identified as an allosteric chaperone for the lysosomal enzyme α-glucosidase [84]. In fibroblasts of patients with Pompe disease, treatment with NAC resulted in significant enhancement of the mutated α-glucosidase activity measured with a fluorescence-based assay using 4-methylumbelliferyl β-D-glucoside. NAC also increased the activity of recombinant human α-glucosidase in fibroblasts, which is the standard-of-care therapy used for this condition [85]. Furthermore, using a computational model of the recombinant human α-glucosidase with NAC, it was shown that NAC promotes an enzyme-stabilizing function at the structural level [86]. These results corroborate in part our findings in Gaucher disease (GD), another lysosomal disorder. In this study, using patient-derived fibroblasts we observed that NAC and its metabolite L-cysteine can act as chaperones and increase the activity of a mutant enzyme. Moreover, using computational modeling, we observe NAC and L-cysteine to bind both active and allosteric sites of this enzyme [87]. Thus, our study shows that these antioxidant chaperones can bind and enhance mutant enzyme activity and can potentially be used to treat patients with GD and, by extension, other related lysosomal disorders caused by misfolded proteins.

## 5. Investigational Uses of NAC in Rare Diseases

In addition to the approved indications of NAC discussed above, there are case reports and clinical studies reported in the literature that explored the potential uses of NAC in a variety of disease conditions.

For instance, the depletion of GSH in aged mice contributes to the impairment of mitochondrial fatty-acid oxidation, which can be re-established by restoring GSH with cysteine and glycine supplementation [88]. A recent randomized clinical trial found that supplementation of NAC and glycine at 100 mg/kg/day each for up to 16 weeks in older adults ameliorates multiple biomarkers, including those related to aging, inflammation, oxidative stress, genomic damage, and mitochondrial dysfunction [89]. Similarly, in retinal pigment epithelium cells from age-related macular degeneration (AMD) donors, NAC protects against oxidative stress by reducing ROS overproduction, GSH and ATP depletion, cell death, and impaired mitochondrial bioenergetics. These findings suggest the therapeutic value of NAC in treating AMD by reducing oxidative damage and mitochondrial dysfunction [90]. NAC is also reported to positively impact neurodegenerative disorders such as AD and PD and mental health disorders through its ability to target mitochondrial impairment [91]. However, the underlying mechanisms of the NAC effect, particularly those unrelated to GSH and targeting mitochondrial dysfunction, are still being investigated [76,92].

In the subsequent sections, we will summarize select (non-exhaustive) literature reports pertaining to the investigational clinical use of NAC in rare genetic disorders, such as primary mitochondrial diseases, and those with associated mitochondrial dysfunction, such as ALS, ALD, Fabry disease (FD), Niemann–Pick disease (NPC), and GD. Specifically, we discuss preclinical studies leading up to clinical trials.

### 5.1. Primary Mitochondrial Diseases

Primary mitochondrial disease is a heterogeneous class of inherited disorders characterized by the deficiency of cellular energy production through oxidative phosphorylation (OXPHOS). There are also secondary mitochondrial diseases that are induced by abnormal processes not related to OXPHOS that can have both inherited and acquired causes, such as aging and inflammation [93], but these will not be covered in this review. Many therapies targeting various physiological processes have been explored for primary mitochondrial disease, including the application of antioxidants to mitigate mitochondrial and cellular oxidative stress [94]. According to the mitochondrial medicine therapies management guidelines published by the Mitochondrial Medicine Society in 2009, NAC has shown promising results as an antioxidant for primary mitochondrial disease in preclinical animal studies [95]. In animal and cellular models of mitochondrial complex I disease, NAC and vitamin E demonstrated greater efficacy than other antioxidants in reducing mitochondrial ROS production, restoring mitochondrial function, and increasing animal survival. However, further research is needed to determine the effectiveness of NAC alone or in combination with other antioxidants as a therapeutic option for primary mitochondrial respiratory chain disease [96]. In TRMU (tRNA 5-methylaminomethyl-2-thiouridylate methyltransferase) deficiency, supplementation with NAC and L-cysteine resulted in clinical improvement in six infants and showed promise in ameliorating the course of this disorder. The infants received supplementation of NAC doses ranging from 70 to 150 mg/kg/day and L-cysteine doses ranging from 85 to 300 mg/kg/day [97]. In addition, NAC was capable of reducing ROS levels and increasing ATP levels in cells from patients affected by adult mitochondrial disease associated with oxidative phosphorylation [98]. Currently, therapeutic strategies, including antioxidants such as analogs of Coenzyme Q10 (CoQ10) and NAC, are under investigation or are close to being in clinical trials for mitochondrial diseases [99].

### 5.2. Rare Diseases with Associated Mitochondrial Dysfunction

Mitochondrial dysfunction has been reported in the pathogenesis of multiple rare diseases, including those due to peroxisomal and multiple organellar dysfunctions, such as ALD and ALS, respectively. Additionally, lysosomal disorders, such as FD, NPC, and GD, are also known to impair mitochondrial function, leading to abnormal cellular processes and contributing to disease progression [100,101].

ROS are essential signaling molecules for healthy cell function. However, excessive ROS production beyond the cellular antioxidant-scavenging capacity can lead to oxidative stress and pathological damage. Superoxide and H_2_O_2_, both examples of ROS, are continuously generated in the mitochondria and participate in signaling pathways between this organelle and others to promote various cellular processes, such as oxidative stress adaptation. The redox state of the electron transport chain (ETC) is one of the major determinants of mitochondrial ROS production. Therefore, an altered redox state of the ETC induced by inhibiting ETC electron carriers can increase superoxide production, contributing to an imbalance in the cellular redox state, which may cause oxidative stress [102,103].

#### 5.2.1. Amyotrophic Lateral Sclerosis

ALS is a rare neurodegenerative disease affecting the central and peripheral nervous systems. The mechanisms that lead to the development of ALS include oxidative stress and mitochondrial dysfunction, and both are part of a broad range of pathophysiologic processes [104]. In preclinical studies examining ALS, the expression of mutant copper/zinc superoxide dismutase-1 in human neuroblastoma cells led to increased mitochondrial ROS and reduced ATP production. Treatment with NAC re-established both mitochondrial function and ROS production to normal levels [105]. Despite these promising findings, a subsequent randomized, double-blind, controlled trial testing NAC administered subcutaneously at 50 mg/kg per day for 12 months in ALS patients failed to change the disease progression parameters, including a decline in muscle strength, pulmonary and bulbar function, disability, and survival rate when compared to the placebo patient group [106]. The dose and the route of administration in this trial were based on previous investigations that showed that subcutaneous administration of NAC at 50 mg/kg dose resulted in concentrations of up to 3 mg/L in the cerebrospinal fluid within 2 to 3 h—hypothesized to provide relief in ALS. However, it is important to note the controlled trial had limited power to detect slight differences in subgroups of patients. Subgroup analysis may suggest a possible benefit of NAC in patients with limb-onset ALS compared to bulbar-onset ALD, whose disease deteriorates more rapidly. Based on these clinical findings and a few additional controlled and uncontrolled studies, NAC has not been recommended as a potential treatment strategy to extend survival in ALS patients [6].

#### 5.2.2. Adrenoleukodystrophy

ALD is a neurodegenerative disorder caused by a mutation in the *ABCD1* gene. This condition results in metabolic abnormalities of very-long-chain fatty acids (VLCFA) and VLCFA-CoA esters, preventing their proper degradation into the peroxisome. As a result of elevated levels of VLCFA, patients with ALD experience adrenal insufficiency and progressive demyelination in both the central and peripheral nervous systems. There are two common phenotypes of ALD: cerebral ALD (cALD), affecting 30–35% of cases (primarily boys), and adrenomyeloneuropathy (AMN), affecting 40–45% of cases, mainly adult men and heterozygous women. The clinical manifestations of ALD are highly variable [107]. The metabolic function of the peroxisome relies on its interaction with various subcellular organelles, including mitochondria, which play a role in re-oxidizing the NADH produced during the peroxisomal beta-oxidation [108]. Morphological abnormalities of the mitochondria have been reported in ALD cell models, mice, and patients [109,110,111], highlighting the contribution of the mitochondria as an underlying pathological mechanism of peroxisomal disorders. The presence of an excess of VLCFA, mainly hexacosanoic acid (C26:0), a substrate of peroxisomal beta-oxidation, in ALD fibroblasts has been found to trigger oxidative stress and decrease the mitochondrial membrane potential [112]. In addition, oxidative damage to components of the mitochondria, such as cyclophilin D, the cytochrome b-c1 complex subunit 2, and the α and β subunits of ATP synthase, may negatively impact the functioning of mitochondria, including the oxidative phosphorylation system. However, the exact mechanism by which saturated fatty acids affect mitochondrial function in ALD remains to be elucidated [113].

In a mouse oligodendrocytic cell model of ALD, NAC was able to prevent the increase in mitochondrial superoxide, depletion of mitochondrial GSH, and impairment in mitochondrial inner membrane potential caused by VLCFA [114]. Moreover, NAC restored GSH homeostasis and reduced lipid peroxidation levels in fibroblasts obtained from skin biopsies from AMN and cALD patients, suggesting a potential for antioxidant-based approaches for treating ALD [115]. Highlighting the significance of oxidative stress in the pathogenesis of ALD, a combination of antioxidants, including NAC, α-lipoic acid, and α-tocopherol was found to alleviate the clinical progression and axonal damage in mouse models of AMN [116]. In an open-label trial involving 13 patients with AMN, a therapy combining high-dose oral NAC, α-lipoic acid, and α-tocopherol decreased most oxidative damage markers and inflammatory markers after three months of treatment [117]. This indicates the protective effect of these antioxidants on the redox imbalance and inflammation status in AMN. NAC is also investigated as an adjunctive therapy along with standard-of-care treatments in cALD. The administration of NAC alone has been shown to enhance the outcomes of hematopoietic stem cell transplantation and decrease oxidative damage in patients with cALD; NAC disposition was also studied in this population [51,118]. New strategies using mesenchymal stem cells, NAC, and eltrombopag have shown favorable outcomes in small clinical trials, stimulating additional studies on a large scale to validate their effectiveness [119].

#### 5.2.3. Fabry Disease

FD is a lysosomal disease linked to a deficiency of the enzyme α-galactosidase resulting from mutations in the *GLA* gene. This leads to an accumulation of globotriasylceramide (Gb3) in lysosomes, resulting in various vascular complications in the kidneys, heart, skin, and brain and a reduced life expectancy. In the US, only one FDA-approved ERT, agalsidase beta (Fabrazyme), is available for treating FD patients [120]. Therefore, identifying additional or adjunctive therapies to ERT would be beneficial for FD patients.

A previous study reported mitochondrial dysfunction in patient-derived FD fibroblasts, marked by reductions in the activities of the respiratory chain complexes I, IV, and V, resulting in decreased cellular levels of energy-rich phosphates [121]. Although ATP levels were slightly changed, the decrease was not significant. Another investigation of the mitochondrial status in peripheral blood mononuclear cells (PBMCs) from FD patients revealed that the lysosomal dysfunction caused by this disease impairs the autophagic function, decreases the ratio of mitochondria/nuclear DNA, and induces the depolarization of mitochondria. Furthermore, in these cells, the mTOR regulation of the ATP synthesis pathway was disrupted. These findings suggest that the inefficient autophagy–lysosomal system exacerbates mitochondrial dysfunction [122]. Additionally, mitochondrial abnormalities may also contribute to lysosomal dysfunction in lysosomal disease [123]. In addition, high levels of ROS production were detected in patient-derived induced pluripotent stem cells (iPSCs) with the *GLA* IVS4 + 919G > A mutation associated with cardiovascular dysfunction, which was significantly decreased by the potent antioxidant effect of NAC. These iPSCs also exhibited rounded and fragmented mitochondria, which can lead to excessive ROS production [124]. These observations are indicative of the beneficial effects of antioxidants in FD that need to be clinically validated.

#### 5.2.4. Niemann–Pick Disease

NPC, type C1 (NPC1) is a lysosomal disorder linked to mutations in the NPC1 gene, leading to the abnormal trafficking and accumulation of lipids, primarily cholesterol, in brain cells. This condition is characterized by a progressive decline in cognitive and motor skills [125]. Previous studies reported elevated cholesterol levels and oxidative stress in NPC1-deficient mice and patients with NPC1 [126,127]. Moreover, NPC1 cell models have shown an inefficient activity of antioxidant enzymes and mitochondrial dysfunction, both of which can contribute to the pathogenesis of the disease [128]. The co-treatment of dermal fibroblasts derived from NPC1 patients with NAC and β-cyclodextrin for 48 h was found to reduce mitochondrial oxidative stress by decreasing the mitochondrial superoxide production [129]. Fu et al. reported modest therapeutic benefits of NAC treatment in both antisense-induced (NPC1ASO) and germline (Npc1−/−) knockout genetic mouse models of NPC1. These results confirm the presence of oxidative stress in the progression of NPC1 and support NAC as a potential treatment option. However, in a clinical intervention of short-term NAC administration in NPC1 patients (NCT00975689), the plasma levels of 7-ketocholesterol, 3β,5α,6β-cholestanetriol, and GSH/GSSG ratio did not change significantly, suggesting a limited efficacy of NAC in the advanced stage of the disease [130]. However, it is important to note that the inadequate efficacy observed could be owing to a suboptimal dose regimen, or inadequate treatment or observation period.

#### 5.2.5. Gaucher Disease

GD is a lysosomal disease caused by mutations in the *GBA1* gene, leading to a deficiency in the activity of the acid β-glucosidase enzyme, also known as GCase. The resulting accumulation of sphingolipids interferes with multiple cellular pathways, contributing to the development of GD pathology. Type 1 GD (GD1) is the most prevalent phenotype and is primarily characterized by non-neurological symptoms, such as hepatosplenomegaly, thrombocytopenia, and bone and lung manifestations. Unlike the other two phenotypes, Types 2 and 3, which are characterized by an early disease onset and progressive neurological symptoms [131], the extent of neurological involvement in GD1 is still not fully understood. Our study, published in 2020, reported neurochemical abnormalities in individuals with GD1 as determined by ultra-high-field magnetic resonance spectroscopy (MRS) measurements [132]. The pathological hallmark of GD includes reduced GCase activity associated with the dysfunction of cellular quality-control pathways leading to mitochondrial dysfunction [101]. This may be associated with inhibiting the macroautophagy pathway, resulting in the impairment of calcium homeostasis, accumulation of α-synuclein, and presence of damaged mitochondria, which are factors that compromise mitochondrial function [133]. Indeed, it has been reported that many patients with GD can develop PD, suggesting an overlap in the underlying pathophysiological mechanisms. Similar to GD, PD is also associated with a damaged mitochondria [134].

The inhibition of GCase activity by using conduritol-β-epoxide (CβE) causes oxidative stress and alters the mitochondrial membrane potential in the human dopaminergic cell model [135]. In a mouse model of nGD, the rate of ATP production in brain mitochondria was significantly lower compared to the control animal [136]. Furthermore, PBMCs obtained from patients with GD showed variation in ATP levels, but there were no statistically significant differences compared to the normal cells. In addition, the lysosomal dysfunction interfered with the autophagy flux and mTOR regulation of ATP synthesis, thus impairing energy metabolism and mitochondrial function [122].

The efficacy of NAC in improving biomarkers and symptoms related to this disease has been evaluated in various cell and mouse models and in clinical trials. In a preliminary Phase I clinical trial (NCT01427517) involving patients with GD1, administering a single IV dose of NAC at 150 mg/kg showed a transient increase in the blood GSH redox ratio and the brain GSH concentrations as measured by 7-Tesla MRS. No adverse effects were observed. These data suggest that NAC has the potential to improve the peripheral and central redox status in GD patients [65]. An interim analysis of an open-label study involving ten patients with GD1 revealed that NAC at a 3600 mg/day dose administered orally for three months showed trends to improve the levels of N-acetyl aspartate and glutamate, which are considered the validated MRS markers for neuronal health, as well as blood antioxidant and inflammatory biomarkers. In addition, improvements in fatigue and alertness were reported by the patients, which may be indicative of improved mitochondrial function [137]. However, limited published studies have explored the effect of NAC on mitochondrial function in the context of GD. Overall, these studies show evidence that NAC can be a potential therapy for all GD phenotypes by minimizing oxidative stress, GCase deficiency, and mitochondrial dysfunction.

### 5.3. Miscellaneous Uses of NAC in Rare Diseases

In addition to the mitochondria-targeted use of NAC, the antioxidant is also being investigated in other rare diseases to alleviate disease-specific symptoms. However, the clinical evidence of its benefits is sparse.

Retinitis pigmentosa (RP) encompasses rare eye conditions affecting the retina, causing vision loss. RP is characterized by rod–cone degeneration, and subsequently, the cones are affected by oxidative stress and undergo cell death. In a Phase I trial, oral NAC was administered at a dose of up to 1800 mg twice and three times a day. The results demonstrated that NAC was safe and well-tolerated during the 24-week treatment period and improved the best-corrected visual acuity of patients with moderately advanced RP. Approximately 30% of patients experienced gastrointestinal adverse events, which resolved spontaneously or with a reduction in the NAC dose [42]. The effects of NAC treatment were explored on locus-level changes in macular sensitivity in RP patients. The findings indicated that NAC at a dosage of 1800 mg twice daily for three months, followed by three times daily for three months, decreased the risk of macular loci sensitivity loss in RP, particularly in the foveal region [138]. Nephropathic cystinosis, an autosomal recessive metabolic disorder, is characterized by intralysosomal cystine storage. It can be managed by therapy with cysteamine, a potent antioxidant, and kidney transplantation. This condition is associated with altered ATP metabolism, deficiency of GSH, and increased susceptibility to oxidative stress. In cystinosis patients treated with cysteamine, NAC at a dosage of 25 mg/kg/day during a three-month period improved renal function and reduced oxidative stress without causing side effects. However, a decline in renal function was observed at three and six months after NAC had been discontinued [139]. Individuals affected by the rare disease known as ryanodine receptor 1-related myopathies (RYR1-RM) present genetic mutations in the RYR1 gene, which plays a crucial role as the main calcium channel in skeletal muscle. The clinical symptoms of RYR1-RM vary in severity and include impaired mobility, muscle weakness, pain, and fatigue. In a clinical trial, participants were treated with oral NAC for six months (adult dose: 2700 mg/day; pediatric dose: 30 mg/kg/day), and the results demonstrated positive outcomes with NAC, particularly in reducing fatigue and weakness. NAC also improved the 6 min walk test, although the results were not statistically significant. In addition, NAC treatment did not significantly change the primary outcome measure for oxidative stress [140]. These trials suggest that NAC may have a positive impact on visual and renal function in patients with RP and nephropathic cystinosis, respectively. Additionally, it has the potential to reduce fatigue and weakness in patients with RYR1-RM. However, further large-scale studies are needed to confirm these findings.

## 6. NAC Derivatives

As evident from the above discussion, the variability in NAC effectiveness may be due to low bioavailability and poor exposure among many other probable causes. In an attempt to circumvent some of those challenges and the lack of NAC’s ability to cross cell membranes to reach the site of action, numerous NAC and cysteine derivatives have been synthesized and tested.

### 6.1. AD4/NACA

N-acetylcysteine amide (AD4/NACA) is a cell-permeant amide form of NAC that has demonstrated superior chemical effectiveness than NAC in various studies. AD4/NACA has been considered a prodrug for NAC since it is rapidly metabolized into NAC after AD4/NACA administration [141]. AD4/NACA reduced oxidative damage and improved both GSH content and mitochondrial bioenergetics in a rat model of traumatic brain injury [142]. Similarly, in a rat model of contusion spinal cord injury, the mitochondrial bioenergetics and GSH content were restored after treatment with AD4/NACA [143]. Moreover, AD4/NACA has high permeability through the cellular and mitochondrial membranes, resulting in elevated bioavailability of this molecule in the CNS [142,144]. In a PK study in mice, AD4/NACA demonstrated a higher GSH-replenishing capacity and bioavailability than NAC due to its effective ability to cross the BBB [145]. A study in a mouse model of multiple sclerosis showed that administering AD4/NACA intraperitoneally or orally effectively suppressed the animal’s clinical signs [146]. Currently, AD4/NACA is undergoing clinical trial in Phase I/II (NCT04355689) for RP associated with Usher syndrome to assess the effect of this molecule on oxidative stress and its efficacy on these diseases.

In summary, multiple studies have shown evidence of the protective effects of AD4/NACA, indicating its potential clinical application for oxidative stress and mitochondrial dysfunction-related diseases [141].

### 6.2. Dendrimer

Dendrimers have been developed to improve drug solubility, bioavailability, and PK and enable targeted drug delivery to specific tissues or cells. Dendrimers are hyperbranched, monodisperse macromolecules that can be used as drug carriers in drug-delivery systems, including oral drug carriers, due to their ability to cross intestinal epithelial cells. This property may increase the permeability and absorption of a co-administered drug when given orally. Dendrimers have also been designed to enhance the drug’s capacity to cross the BBB [147,148]. For instance, sulfadiazine complexed with fourth-generation polyamidoamine (PAMAM) dendrimers administered via IV showed a tenfold higher concentration of this drug in the brain, and low toxicity was observed in rats [149]. A delivery system for NAC was tested by conjugating a well-known mitochondrial targeting agent, triphenyl-phosphonium (TPP), with NAC to the surface of hydroxyl PAMAM dendrimer in murine microglia cells. TPP-conjugated dendrimer NAC effectively attenuated mitochondrial oxidative stress and protected cells from oxidative-stress-induced cell death [150]. Furthermore, administration of TPP-conjugated D-NAC in a mouse model of AMD induced a significant reduction in mitochondrial ROS in retinal pigment epithelial cells, indicating a novel potential therapy to explore for AMD, which has no effective cure [151]. In ALD, a dendrimer conjugated with NAC (D-NAC) demonstrated greater efficacy than NAC alone in improving the immunological response. Extracellular free VLCFAs (C24:0 and C26:0) induced tumor necrosis factor α and glutamate secretion and depleted the total GSH in cALD. This was completely reversed by D-NAC in macrophages from patients with cALD, while glutamate secretion was reduced in AMN cells [152]. Additionally, D-NAC was shown to be more effective than NAC in bypassing the cysteine–glutamate antiporter (Xc-) for cell uptake, increasing the GSH levels while preventing both glutamate release and excitotoxicity in mixed glia derived from Mecp2-null mice, a model of Rett syndrome. Furthermore, D-NAC improved behavioral outcomes but not survival in these mice. These findings suggest that the delivery of drugs using dendrimers could be a potential strategy for targeting glia and attenuating oxidative stress and immune dysregulation in patients with Rett syndrome, a rare progressive developmental disorder with no effective cure [153]. The dendrimer drug-delivery platform can also be applied to other neurological conditions requiring targeted glial treatment [153,154].

### 6.3. NAC Ester

N-acetylcysteine ethyl ester (NACET) is produced through the esterification of the carboxyl group of NAC to improve its lipophilicity, bioavailability, and efficacy. In a rat model, orally administered NACET has been shown to have the ability to enter cells and produce NAC, cysteine, and H_2_S, a critical gaseous signaling molecule. Compared to NAC, NACET-derived NAC demonstrated higher PK profiles and greater GSH content in different tissues and the brain. Therefore, NACET can be a more effective GSH precursor for increasing GSH in stressed cells. NACET also holds promise as an antioxidant for many therapeutic applications, such as paracetamol overdose intoxication [155]. Moreover, N-acetylcysteine butyl ester (NACBE), a highly lipophilic NAC prodrug, showed a superior effect in increasing the mitochondrial GSH content and protecting against mitochondrial membrane depolarization and ATP loss after an oxidative insult exposure when compared to NAC in human retinal pigment epithelial cells [156].

### 6.4. Thiazolidines

Thiazolidines (e.g., 2-oxothiazolidine-4-carboxylic acid, Cys-OTC) are a type of cysteine product that is transported and enters most cells and is rapidly converted into cysteine. Cys-OTC has been reported to have a moderate effect on increasing GSH levels, and it has also been shown to be effective in reversing endothelial dysfunction in patients with coronary artery disease [157].

## 7. Discussion and Conclusions

In this review, we summarized the available literature on NAC pharmacology and mechanism of action to provide a context for its investigational use in several rare diseases.

It is well-appreciated that several clinical and clinical pharmacology challenges are encountered in rare disease drug development. The smaller rare disease patient population, and very often the heterogeneity of disease progression seen with many rare diseases, can seriously impact the ability to establish evidence of effectiveness using conventional trial designs. Clinical pharmacology studies are often undertaken for appropriate dose selection; the dose is optimized based on intrinsic or extrinsic factors such as age, genotype, gender, co-morbidities, etc. Repurposing an approved treatment such as IV or oral NAC for a rare indication is not free of challenges, as the intrinsic and extrinsic factors guiding dose optimization can be very different for the newly proposed indication. In cases where the drugs are repurposed from previously approved rare or non-rare indications and where robust information on the safety and mechanism of action exist, dose selection for the new indication can be informed by the previously approved doses for other indications. A similar approach was explored for NAC; for example, IV NAC at 70 mg/kg is approved by the FDA as a maintenance dose for acetaminophen overdose [23]. IV NAC at the same dose was used as a supporting regimen before and after hematopoietic stem cell transplant for pediatric patients of inherited metabolic disorders. In addition to the lack of registration enabling studies, as NAC is available OTC and the physicians can choose to prescribe it off-label, it is likely that there will not be formal approvals of NAC for newer indications.

NAC was first approved in the 1960s for its use in muco-pulmonary and antidote indications for which the primary outcome measures were focused on clinical improvement. Routine clinical pharmacology studies such as SAD, MAD, mass-balance, and DDI studies were not performed in the case of NAC approvals, limiting the available information on the drug, which has been extensively used since the 1960s. This allows us to reflect upon clinical pharmacology and regulatory sciences development over the past 60 years. Since its approval, NAC has also been used as an OTC dietary supplement. NAC has since been explored for a multitude of diseases, ranging from hepatic and renal diseases to cancer and HIV.

Through pre-clinical studies, many mechanisms of action have been attributed to NAC; some are better-elucidated than others. However, the clinical effect has often been variable and conflicting [158], even for the pulmonary complications for which NAC is approved [20]. Although the exploratory applications of NAC use are widespread, there are reports of the ineffectiveness [106,130] of NAC, whereas some other studies point toward probable effectiveness [44,158,159]. The publication bias also needs to be considered in weighing the evidence. None of these explorations have led to FDA approval for any of the new indications. NAC is used as a dietary supplement by many, despite systematic efforts to elucidate its benefit being lacking. The differences in the regulation of dietary supplements vs. prescription drugs could be another contributing factor.

Orally administered NAC is hydrolyzed by deacetylases into cysteine. NAC exists as a variety of moieties in vivo. There are challenges associated with the bioanalytical quantitation of cysteine, as it is an endogenous molecule that is also abundant in some foods. The complexity of an assay mounts further if simultaneous detection of NAC and cysteine is desired. For instance, due to the poor bioavailability of NAC, the systemic levels following oral NAC doses are in the nanogram to microgram range, whereas, due to the high conversion of orally administered NAC to cysteine in addition to endogenous levels, the concentrations of cysteine are in the microgram to milligram range. The paradox of needing amplification of the signal for NAC and needing dilution of the sample to suit the signal-detection limits for cysteine is challenging for any analytical chemist. This difference in the orders of magnitude poses assay-development challenges ranging from selecting an adequate sample volume for the assay to the optimization of reagent concentrations and sample-separation and -detection settings. While a simultaneous assay can save time and the cost of sample analysis, the challenges of assay development and validation may make that an infeasible choice.

Based on our experience, a hurdle in the expanded clinical use of NAC is that there is no clear characterization of what constitutes a “response” to NAC in patients with different diseases. Based on the proposed mechanisms of NAC action, we measured changes in the brain or blood GSH or redox ratio, peripheral measures of inflammation as a response to NAC treatment; however, whether such a change represents symptomatic relief or if it influences the rate of disease progression or a combination of the two is unknown. That would be ideal if NAC could alter the disease course while providing symptom relief. A clinical study to track the rate of disease progression with and without NAC treatment would need a longitudinal follow-up spanning years (e.g., for rapidly progressing disorders such as cALD) or decades (e.g., for slower-progressing conditions such as GD1 or AMN). Ethical approval for such a study would also be a challenge. The oxidative stress and inflammation biomarkers can be looked at as surrogate markers of the ultimate clinical response. Investigations to elucidate the missing links between the PK, surrogates of response, and ultimate clinical response have the potential to increase the direct impact of the literature reviewed in this report. Moreover, the treatment of rare diseases may necessitate the chronic use of NAC. In that sense, it is important to note that high doses of NAC have been reported to induce cancer metastasis [160]. Caution needs to be exercised against the uncontrolled use of NAC or other similar drugs.

As many rare diseases are related in their underlying pathophysiology and symptomatology, explorations of NAC in one rare disease can be reasonably and cautiously extrapolated to similar rare diseases, and we attempt to facilitate such extrapolations and advancements through this literature review. The expansion of antioxidant gene therapies highlights the critical role of antioxidant defenses in the maintenance of health [161,162]. As cell and gene therapies inch closer to transitioning from trials to clinical practice for the treatment of rare diseases (e.g., GD or cALD), avenues to enhance their benefit through the correction of oxidative stress may open new directions for research with NAC or its derivatives as an adjunct therapy [163].

## Figures and Tables

**Table 2:** NAC PK parameters.

Study	Dose	Form	Mean t½ (h)	Mean CL (or CL/F) (L/h/kg)	Mean V (or V/F) L/kg	Model/Notes
Börgstrom et al., 1986 [31] 10 adult HV	600 mg IV 600 mg oral	Non- protein-bound NAC	2.27 (IV) (elimination)	0.211	0.33	Bioavailability 6–10%
Olsson et al., 1988 [32] 6 HV	200mg IV 400 mg oral	Total NAC	5.58 (IV) (terminal)	0.19	0.47	Total NAC concentrations declined in a triphasic manner. Bioavailability 9.1%. From one hour onward, covalent protein binding of NAC increased, reaching maximum 50% at 4 h, and decreased to 20% at 12 h post-dose
Prescott et al., 1989 [33] 17 patients of acetaminophen overdose	150 mg/kg IV over 15 min followed by 50 mg/kg IV in 4 h, and 100 mg/kg over 16 h	Total NAC	5.7 (±2.9) (terminal)	0.19	0.54	
Ahola et al., 1999 [34] 10 preterm infants	4.2 mg/kg/h for 24 h (continuous IV infusion)	Total NAC	11 (elimination)	0.037	0.57	
Weist et al., 2014 [35] 11 pregnant women 5 preterm infants 6 near-term infants	100 mg/kg IV q4h 12.5 mg/kg q12h 25 mg/kg q12h	Total NAC	1.2 7.5 5.1	0.26 0.045 0.07	0.41 0.47 0.34	
Coles et al., 2017 [36] 4 patients with PD 3 HV	Steady-state PK following 3000 mg oral NAC	Total NAC	4.6 5.9	66.6 L/h	269 L	NCA used for t½. Pop PK model first-order absorption, 1-compartment, proportional error model (estimated PD, HV together)
Papi et al., 2021 [37] 15 HV Chinese 15 HV Caucasian	Oral effervescent tablet, NAC 600 mg first as a single dose and, following a 48 h wash-out period, twice daily for 3 days.	Total NAC	15.4 ± 3.5 18.7 ± 7.2	1250.0 ± 474.9 1400.8 ± 508.5	56.9 ± 16.2 56.0 ± 20.1	Estimated Chinese and Caucasian separately. Accumulation ratio Chinese 1.5 ± 0.4 and Caucasian 1.4 ± 0.2. V, CL, t½ estimated after single dose, expressed as mean ± SD
Greene et al., 2016 [38] 29 HV effervescent NAC. 30 HV NAC solution 11g NAC oral dose in both periods	A single-dose, randomized-sequence, 2-period crossover design with a 7-day washout period	Total NAC	18.1 ± 3.96 17.5 ± 2.98	65.1 ± 22.8 59.3 ± 16.3	1720 ± 731 1510 ± 503	NCA µg/mL Cmax (oral solution, effervescent tablet, resp.) 28.4, 26.5 µg/mL, resp. Estimates of V/F are in L (not normalized to weight). Relative F = 94 ± 18.5 effervescent/solution ratio of AUCinf values × 100.
Liu et al., 2010 [39] 24 HV Chinese adults 3 × 200 mg test effervescent NAC. 600 mg effervescent oral NAC (reference: Fumicil®)	A single-dose, randomized-sequence, 2-period crossover design with a 7-day washout period	Total NAC	6.07 ± 2.41 5.62 ± 2.60	-- --	-- --	NCA

Abbreviations: HV, healthy volunteers; PD, Parkinson’s disease; IV, intravenous; Pop PK, population pharmacokinetics; NCA, non-compartmental analysis; AUC, area under the curve; SD, standard deviation.
